# Nutritional modulation of microbiome proteomics in insulin resistance

**DOI:** 10.3389/fnut.2026.1913314

**Published:** 2026-10-21

**Authors:** Beraat Dener, Sueda İzem Gülal, Miraç Bulut, Hilal Hızlı-Güldemir, Bence Raposa, Duygu Aǧagündüz

**Affiliations:** 1 Department of Nutrition and Dietetics, Faculty of Health Sciences, Bingöl University, Bingöl, Türkiye; 2 Department of Nutrition and Dietetics, Institute of Health Sciences, Aydın Adnan Menderes University, Aydın, Türkiye; 3 Department of Nutrition and Dietetics, Faculty of Health Sciences, Demiroǧlu Bilim University, İstanbul, Türkiye; 4 Department of Nutrition and Dietetics, Faculty of Health Sciences, Anadolu University, Eskişehir, Türkiye; 5 Institute of Basics of Health Sciences, Midwifery and Health Visiting, Faculty of Health Sciences, University of Pécs, Pécs, Hungary; 6 Department of Nutrition and Dietetics, Faculty of Health Sciences, Gazi University, Emek, Ankara, Türkiye

**Keywords:** G-Protein-coupled Receptor 43 (GPR43), insulin resistance, metaproteomics, proteome layer, proteomics

## 1 Introduction

The gut microbiota serves as a pivotal mediator of host energy homeostasis, influencing metabolic health by regulating systemic inflammation and secreting bioactive metabolites (1). Recent multi-omics investigations have identified specific shifts in microbial carbohydrate metabolism that correlate with increased systemic monosaccharide levels and the subsequent induction of inflammatory cytokines (2, 3). Specifically, the modulation of microbial enzymatic pathways through targeted dietary interventions offers a therapeutic avenue to dampen endotoxemia and improve peripheral insulin sensitivity (4, 5). Moreover, interindividual baseline variations in gut microbial functionality significantly dictate the host's metabolic response to macronutrient perturbations, underscoring the necessity of precision-based nutritional strategies (6). Rethinking these dynamics through a meta-organism lens suggests that insulin resistance should be framed as a systemic metabolic dysregulation wherein functional pathways—rather than mere taxonomic composition—become the primary drivers of host health (3, 7). Therefore, this study aims to review the nutritional modulation of microbiome proteomics in insulin resistance. It also examines the matter of proteome layer in insulin resistance, metaproteome, host-microbiome protein crosstalk and precision management of insulin resistance toward nutrition centered metaproteomic framework. The literature review was conducted between May 4, 2026, and July 1, 2026. Studies published after 2010 were included in the text after searching the PubMed, Web of Science Core Collection, Scopus, and Cochrane Library databases using the keywords “insulin resistance,” “diabetes,” “microbiome,” “microbiota-host interactions,” “protein-protein interactions,” “insulin signaling,” “insulin resistance,” “proteomics,” “proteome layer,” “metaproteomics”. In addition Google Scholar, ResearchGate, and ScienceDirect were used as supplementary sources to identify potentially relevant studies and minimize the risk of missing relevant literature. The artificial intelligence tools NotebookLM and Elicit were additionally used to support the identification and screening of relevant records. In addition, the reference lists of the included key articles and relevant reviews were retrospectively searched. Studies were included if they examined the relationship between diet, food or food components and changes in the microbiome proteome, or if they linked these changes to insulin resistance or sensitivity. Human studies (RCTs, prospective cohorts, cross-sectional studies), animal studies, protocol studies, and mechanistic *in vitro* studies were included. Publications not in English, content for which the full text was not accessible, conference abstracts, opinion articles that did not present data, and studies addressing off-topic populations (e.g., other metabolic diseases) were excluded. The retrieved findings were first prioritized according to a methodological/design hierarchy. Each key claim in the text was then clearly assigned to one of three categories: Those providing strong evidence, such as which supported by consistent findings from multiple human studies and/or independent mechanistic validation; those providing moderate evidence, such as a limited number of human studies or a single robust design; those providing preliminary or hypothesis-generating evidence, such as cross-sectional studies, small-sample studies, or preclinical data alone.

## 2 Why the proteome layer matters in insulin resistance

Insulin resistance (IR) is a fundamental pathological mechanism at the heart of metabolic disorders such as type 2 diabetes (T2D), obesity, and gestational diabetes mellitus (GDM). The proteome layer, which characterizes proteins as the primary functional units of the cell, is essential for deciphering these mechanisms since proteins play a fundamental role in regulating and catalyzing the majority of cellular processes. While transcriptomic studies provide valuable insights into gene expression, the frequently low correlation between transcript and protein levels necessitates proteomic analysis to achieve true biological accuracy (8). Proteomics enables the direct measurement of protein abundance, post-translational modifications (PTMs), and the secretome, all of which contribute significantly to determining protein function and biological action (9). Skeletal muscle, responsible for approximately 80% of insulin-stimulated glucose disposal, is the primary site of peripheral insulin resistance (9). Drawing largely from preclinical models and early-stage human investigations, proteomic research in this tissue indicates that IR conditions are frequently associated with a reduced abundance of mitochondrial oxidation proteins and alterations in the phosphorylation of critical complexes, such as the ATP synthase beta-subunit (10). These disruptions in the mitochondrial proteome may significantly contribute to impaired energy metabolism at the tissue level and potentially weaken insulin signaling pathways (10).

Furthermore, systematic reviews of obesity reveal that the proteome layer reflects an increase in proteins associated with inflammation and oxidative stress, such as complement C3 and catalase (11, 12). In the context of GDM, proteomic meta-analyses have identified 262 candidate biomarkers, most strongly linked to pathways in the complement and coagulation cascades (13, 14). Ultimately, proteomic data can help establish a vital foundation for the vision of Precision Diabetes Medicine (PDM) (8). By providing precise protein panels, this layer enables personalized diagnostic and therapeutic strategies that account for individuals' unique genetic and environmental differences (8).

## 3 The metaproteome as a functional readout of insulin-resistant dysbiosis

Metaproteomics serves as a powerful “functional readout” tool by identifying the actual proteins expressed by the gut microbiota and the host, providing insights into active biochemical processes that traditional taxonomic sequencing cannot capture (15). In metabolic disorders such as insulin resistance (IR) and type 2 diabetes, the functional shifts in the microbial proteome often reflect the disease state more accurately than changes in microbial composition alone (15). While 16S rRNA sequencing captures the potential of a microbial community, metaproteomics captures its dynamic plasticity and its response to environmental stressors such as diet, inflammation, or medication (15, 16).

In the context of insulin-resistant dysbiosis, the metaproteome reveals significant shifts in carbohydrate and energy metabolism (15). Obese and insulin-resistant individuals frequently exhibit an upregulation of microbial proteins involved in starch and pectin metabolism, such as β-glucosidase and pectate lyase (15). This enrichment suggests an enhanced microbial capacity to ferment unabsorbed carbohydrates into bioavailable short-chain fatty acids (SCFAs), which may provide the host with an additional energy source and exacerbate metabolic dysfunction (15, 17). Furthermore, specific enzymes, such as butyryl-CoA dehydrogenase, are often differentially expressed in IR states, potentially influencing signaling pathways, such as PI3K/Akt, that regulate systemic insulin sensitivity (16, 18).

Childhood obesity and IR studies further illustrate the utility of this approach, showing that reductions in beneficial species, such as Akkermansia muciniphila, correlate with decreased levels of host serum proteins involved in metabolism and immunity, such as IGFBP1 and GP6 (19). The metaproteome also documents the disruption of the intestinal barrier by tracking changes in host structural proteins, such as mucin-2 and various immunoglobulin polypeptides, which serve as biomarkers for low-grade inflammation and increased gut permeability (15, 20). Ultimately, proteomic data contribute a valuable conceptual framework for the vision of Precision Diabetes Medicine (PDM) as an evolving translational model (8). Rather than an immediately applicable clinical tool, this framework currently serves as a conceptual model—requiring extensive clinical validation before precise protein panels can be routinely integrated into personalized diagnostic and therapeutic strategies.

## 4 Host–microbiome protein crosstalk- linking nutritional signals to insulin signaling pathways

The symbiotic relationship between the host and its colonizing microbiota operates as a complex “metaorganism,” wherein inter-kingdom protein-protein interactions modulate fundamental signaling networks and physiological processes (21). This highly evidence-based recent research, host-focused analysis, which comprehensively adds human functional annotations to commensal bacterial proteins, indicates that this crosstalk is mediated by targeting human proteins involved in critical immune, oncogenic, apoptotic, and endocrine pathways, notably those linked to the development of insulin resistance (22). More directly, a brief new study which commensal Pseudomonadota isolated from the human gut reported that commensal type III secretion effectors can translocate into human cells and modulate NF-κB signaling and cytokine secretion *in vitro* (23). Specifically, in a protocol study that could be considered to have more limited results this communication often occurs via domain-motif interactions that facilitate the integration of microbial effectors into human molecular networks (24). In these configurations, microbial proteins specifically target motifs on host proteins to drive post-translational modifications, effectively reconfiguring cellular homeostasis and modulating pathways linked to insulin resistance (3, 22).

At the protein level, the clearest links to insulin resistance run through innate immune receptors and barrier proteins rather than a large catalog of named host–microbe protein pairs. The cleanest pathway is bacterial cell-wall sensing. Thus, this relationship is bidirectional. According to an animal study that could be considered a preliminary finding is that acute insulin resistance itself causes gut hyperpermeability, dysbiosis, and a collapse of antimicrobial defenses, and they conclude that insulin signaling is “an indispensable gatekeeper of intestinal barrier integrity” (20). At a mechanistic level, nutritional signals feed into insulin signaling through two overlapping channels: the nutrients themselves and the metabolites they generate. Smith and colleagues report that GPR43 senses SCFAs and regulates adipose insulin signaling, which is one of the clearest protein-level links between diet-derived microbial products and insulin action in mice (25).

In summary, the relationship is not a single linear pathway. It is a signaling web: diet changes microbial metabolism, microbial metabolites hit host receptors, and those inputs rewire insulin action in the intestine, liver, adipose tissue, and muscle. Yet, a more targeted follow-up study could separate human evidence from animal work or map specific ligand–receptor pairs in more detail.

## 5 Current methodological constraints in nutritional metaproteomics

Current methodological limitations restrict the potential of nutrition-focused metaproteomic studies in insulin resistance research. Although metaproteomics provides functional information, analytical and computational challenges reduce reproducibility. The influence of diet, medication, and microbiota diversity on gut and fecal samples makes it difficult to identify biomarkers associated with insulin resistance (26, 27). Another important limitation of the method is the sample preparation process. Variability in protein extraction protocols, storage conditions, and peptide preparation methods can reduce the recovery of usable proteins and decrease the comparability between studies. In particular, the simultaneous presence of microbial, host, and food-derived proteins in gastrointestinal samples introduces significant analytical complexity. The high abundance of host proteins can mask the detection of lower-abundance microbial proteins involved in glucose metabolism, inflammatory regulation, and insulin hormone stimulation (28, 29). Additionally, differences in microbial taxa and cellular compartments can influence protein extraction efficiency, resulting in differential recovery of microbial proteins during sample processing (30).

Limited proteome coverage and incomplete microbial databases restrict mass spectrometry-based analyses. Many gut microorganisms are poorly characterized, leading to ambiguous protein identifications and insufficient functional annotation. These issues reduce taxonomic accuracy and make it difficult to identify diet-sensitive microbial pathways associated with insulin resistance (31, 32). Although new sequencing strategies have been proposed to overcome these limitations, higher accuracy and computational efficiency remain required (33). Differences in bioinformatics and the lack of standardized methods reduce data reproducibility. Additionally, integrating metaproteomic, metagenomic, metabolomic, and transcriptomic data presents significant computational challenges (33, 34). Additionally, nutritional intervention studies are limited by short follow-up periods, inconsistent dietary assessments, and inadequate control of factors such as obesity, physical activity, and medication use (35, 36). In conclusion, strengthening methodological standardization and improving microbial databases are essential for translating metaproteomic findings into precision nutrition practices for insulin resistance.

## 6 Toward a nutrition-centered metaproteomic framework for precision management of insulin resistance

Insulin resistance is a complex condition resulting from the interaction of microbial functions, nutrition, and host metabolism. Changes in microbial functions may play a key role in insulin resistance and type 2 diabetes. Metaproteomics is promising for identifying microbial proteins that influence glucose metabolism and inflammation (2, 3). Unlike metagenomics, metaproteomics not only reveals microbial taxa and genetic potential, but also allows for the assessment of microbial activity under specific nutritional conditions. This distinction is important because diet has the potential to rapidly alter microbial protein expression and metabolite production. Several studies investigating resistant starch and dietary modulation have suggested that alterations in the dietary nutrient intake of healthy individuals may affect microbial enzymes involved in carbohydrate metabolism and the synthesis of short-chain fatty acids (37, 38). Advances in personalized nutrition highlight the importance of nutrition-focused metaproteomic approaches in insulin resistance. Since individuals respond differently to dietary interventions, standard recommendations may not always be effective. Therefore, personalized strategies based on microbial functions, host metabolism, and inflammatory markers can provide more targeted outcomes (39, 40). In addition, microbial proteins involved in lipopolysaccharide biosynthesis, oxidative stress, branched-chain amino acid metabolism, and bile acid transformation may be linked to impaired insulin signaling and chronic inflammation. Metaproteomics can help identify biomarkers that predict dietary response and metabolic risk at an early stage (41, 42).

Nutrition-focused metaproteomics can support personalized nutrition strategies in insulin resistance. Since individuals show different metabolic responses to the same dietary interventions, personalized approaches are needed (39, 40). Addressing these factors collectively may facilitate the targeted modulation of microbial functions, improve insulin sensitivity, and reduce the risk of metabolic diseases, while providing a foundation for further research into personalized nutrition and metaproteomic approaches.

## 7 Discussion

The aforementioned findings demonstrate that insulin resistance transcends a single tissue or taxon, emerging instead from the complex interplay among diet, the microbiota, and the host proteome. Findings at the proteome level reveal the limitations of transcriptome data, showing that researchers can capture true biological activity only through investigations of protein abundance and post-translational modifications. The discovery of a “core metaproteome”—comprising 182 microbial functions and 83 metabolic pathways shared across all individuals, identified through the reanalysis of ten fecal metaproteomic datasets from healthy subjects—further corroborates this concept (43). This suggests that the metaproteome provides a functional readout plane entirely independent of taxonomic diversity.

Analyzing mitochondrial proteome alterations in skeletal muscle alongside the upregulation of carbohydrate-metabolism enzymes in the gut metaproteome suggests that peripheral insulin resistance may be linked to a simultaneous “energy surplus” state in both the host and the microbiome. A human multi-omics study demonstrates elevated fecal monosaccharide levels in insulin-resistant individuals, which directly correlate with microbial carbohydrate metabolism and host inflammatory cytokines (3).

Host-microbiome crosstalk appears to establish, a bidirectional feedback loop: while these interactions maintain gut barrier integrity, any barrier disruption indirectly impairs insulin signaling based on preliminary animal evidence. A comprehensive review examining the microbiota-carbohydrate metabolism axis and microbiota-host interactions positions these domains as central to understanding the microbial mechanisms driving insulin resistance; however, the literature indicates that most relationships outside the GPR43-SCFA axis still rely exclusively on animal models (44).

Methodological challenges regarding sample preparation, database coverage, and multi-omics integration in the aforementioned studies explain why the summarized mechanistic findings currently remain largely correlational. Host protein dominance masks low-abundance yet functionally critical microbial proteins (such as enzymes that modulate insulin signaling), thereby directly limiting biomarker discovery. This limitation underscores the urgent need for individualized and standardized protocols in nutrition-focused metaproteomic approaches. A metagenome-informed metaproteomic study in mice and humans demonstrates that compositional dysbiosis and species-specific functional dysbiosis manifest simultaneously during dietary shifts; this strategy provides a highly promising methodological model to overcome the detection limits of low-abundance, functionally critical microbial proteins (45) (Figure 1).

**Figure 1:**
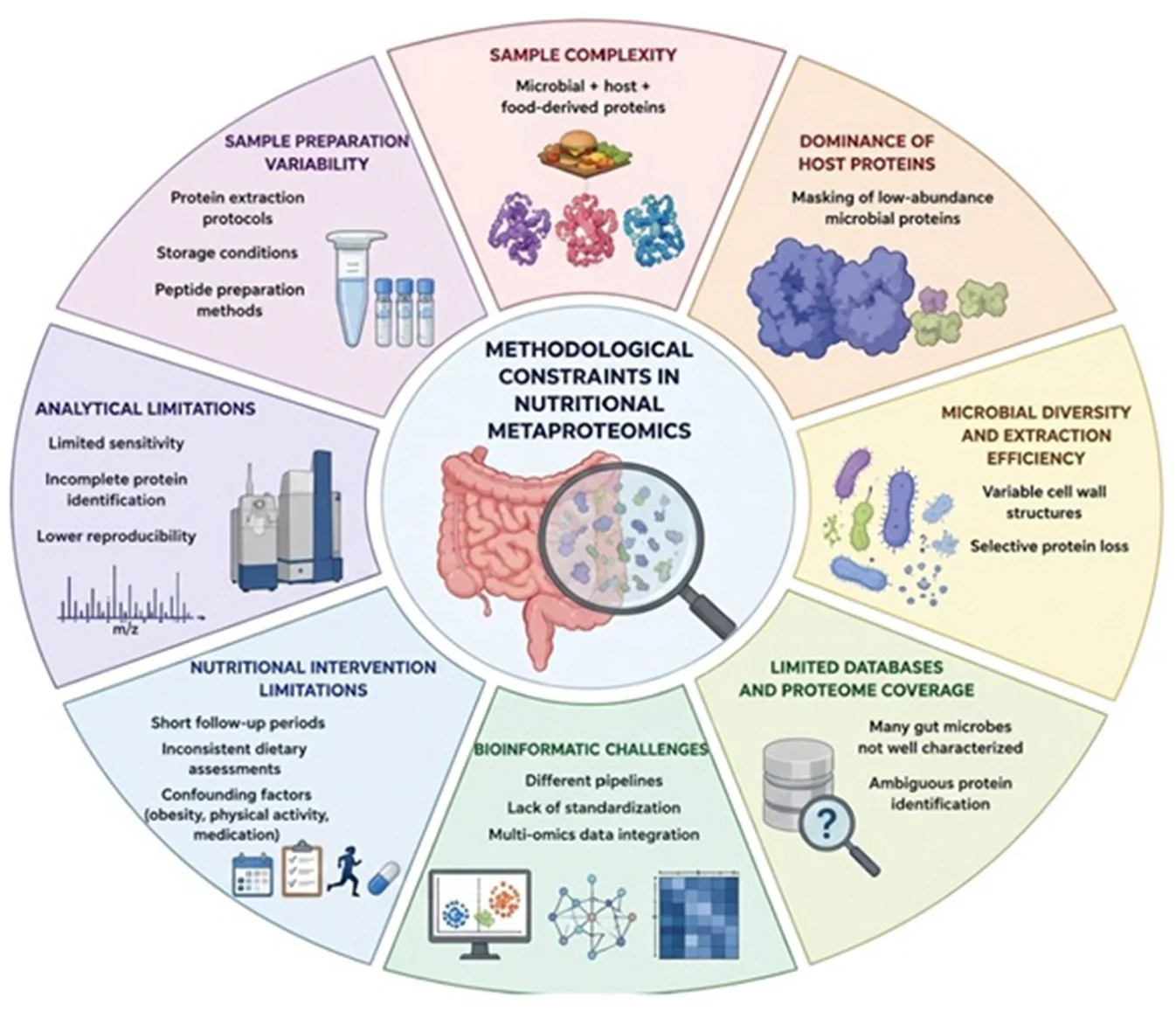
Current methodological constraints in nutritional metaproteomics.

Finally, the proposed personalized nutrition framework offers a logical synthesis that integrates all the aforementioned layers: fundamental interpersonal differences in microbial function drive the varied metabolic responses to identical dietary interventions. Consequently, evaluating metaproteomic data alongside diet, microbiota function, and host inflammatory markers may, in the longer term and pending extensive clinical validation, contribute to the still-evolving conceptual vision of materializing the vision of Precision Diabetes Medicine along the gut axis.

Interactions between host and microbiota proteins are appear to linked, though lergely on the basis of animal and *in vitro* evidence to insulin signaling, particularly through gut barrier integrity, short-chain fatty acid receptor signaling, and inflammatory pathways. However, completing this mechanistic map requires strict methodological standardization across sampling, database coverage, and data integration.

We propose three priority areas for future research: (1) longitudinal monitoring of metaproteomic shifts before and after dietary interventions in human cohorts; (2) validation of microbial protein-host receptor interactions within human tissues, specifically emphasizing GPR43-like pathways; and (3) the development of predictive models for dietary responses by integrating metaproteomic, metagenomic, and metabolomic data at the individual level. Advancements in these areas will lay the critical groundwork for translating microbiota-targeted, personalized nutrition strategies into clinical practice to prevent and manage insulin resistance.
